# The exocyst in context

**DOI:** 10.1042/BST20231401

**Published:** 2024-10-08

**Authors:** Sasha Meek, Altair C. Hernandez, Baldomero Oliva, Oriol Gallego

**Affiliations:** Department of Medicine and Life Sciences, Universitat Pompeu Fabra, Barcelona 08003, Spain

**Keywords:** evolutionary cell biology, exocyst, exocytosis, structural cell biology

## Abstract

The exocyst is a hetero-octameric complex involved in the exocytosis arm of cellular trafficking. Specifically, it tethers secretory vesicles to the plasma membrane, but it is also a main convergence point for many players of exocytosis: regulatory proteins, motor proteins, lipids and Soluble N-ethylmaleimide-sensitive factor Attachment Protein Receptor (SNARE) proteins are all connected physically by the exocyst. Despite extensive knowledge about its structure and interactions, the exocyst remains an enigma precisely because of its increasingly broad and flexible role across the exocytosis process. To solve the molecular mechanism of such a multi-tasking complex, dynamical structures with self, other proteins, and environment should be described. And to do this, interrogation within contexts increasingly close to native conditions is needed. Here we provide a perspective on how different experimental contexts have been used to study the exocyst, and those that could be used in the future. This review describes the structural breakthroughs on the isolated *in vitro* exocyst, followed by the use of membrane reconstitution assays for revealing *in vitro* exocyst functionality. Next, it moves to *in situ* cell contexts, reviewing imaging techniques that have been, and that ideally could be, used to look for near-native structure and organization dynamics. Finally, it looks at the exocyst structure *in situ* within evolutionary contexts, and the potential of structure prediction therein. From *in vitro*, to *in situ*, cross-context investigation of exocyst structure has begun, and will be critical for functional mechanism elucidation.

## Structural biology: unveiling the isolated exocyst

The exocyst is a hetero-octameric protein complex canonically involved in the tethering of secretory vesicles (SVs) to the plasma membrane in constitutive exocytosis (hereafter exocytosis). The importance of exocytosis is far-reaching within the cell, being essential for cell growth, plasma membrane homeostasis, polarization and motility, among many more [1,2]. Consequent with this essentiality, the exocyst is conserved across most eukaryotes [3]. The eight exocyst subunits were first described in yeast [4–6], thereafter in metazoans [7,8] and in higher plants [9]. The exocyst is structurally organized in two tetrameric modules: Sec3, Sec5, Sec6, and Sec8 subunits form the first and Sec10, Sec15, Exo70 and Exo84 subunits form the second [10,11]. Each module is held together by the association of four coiled coil (CC) motifs (alpha helices ranging between 67 and 115 amino acids), one from each subunit, forming the Core of Exocyst (CorEx) motif [10,11].

**Figure 1.**
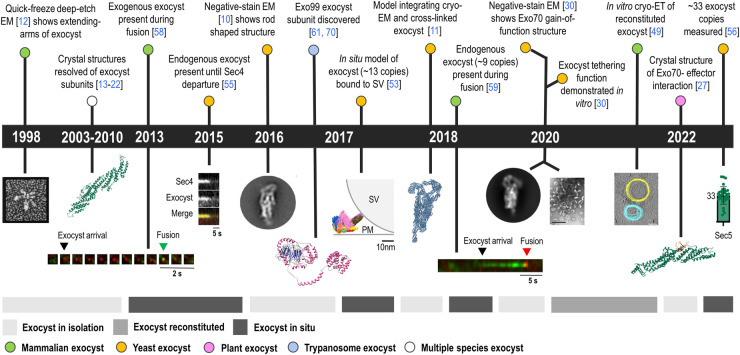
Chronological timeline (∼25 years) showing some of the main insights into the exocyst's structure. *In vitro* and *in situ* structural analysis, live-cell dynamics and evolutionary differences of the exocyst complex show the cross-context scales in which it has been studied. The gray bars below the timeline state these contexts. Figures adapted from (left to right): [10,12,30,49,53,55,56,58,59,61]. The yeast Exo70 crystal structure (PDB ID: 2B7M, [18]), yeast cryo-EM model (PDB ID: 5YFP, [11]) and rice plant Exo70F2 in complex with AVR -Pii effector crystal structure (PDB ID: 7PP2, [27]) were accessed from the PDB.

A concerted effort across three decades has led to the elucidation of the nearly-full exocyst complex structure (Figure 1). In 1998, quick freeze deep-etch electron microscopy (EM) of a glutaraldehyde-fixed whole mammalian exocyst showed a low-resolution structure: a compact body with extending arms [12], suggesting some flexibility (Figure 1). Crystal structures of regions of individual subunits were then solved between 2003 and 2010 (Figure 1): mammalian Sec5 [13], Exo70 [14] and Exo84 [15,16]; yeast Exo70 [17,18], Exo84 [17], Sec6 [19] and Sec3 [20,21]; and *Drosophila* Sec15 [22]. Later, from 2017 to present, mammalian Sec8 [23], yeast Sec3 [24], zebrafish Sec10 [25] and plant Exo70 [26,27] were resolved. The subunits, mostly their C-terminal regions, were found to have multiple alpha-helical bundles, organized in tandem repeats [28]. This structural feature motivated the classification of the exocyst within the complexes associated with tethering containing helical rods (CATCHR) family [29]. Alpha-helical bundles result in rod shaped structures, whose interaction interface with other subunits lie along the length of the rod. Negative stain EM revealed a laterally packed rod structure of the full complex [10] and an Exo70 gain-of-function mutant [30] (Figure 1). The yeast exocyst in isolation was modeled in 2018 [11] by integrating a 4.4 Å resolution cryo-EM structure of the nearly-full length exocyst with cross-linking mass spectrometry and homology modeling. (Figure 1). The CorEx of each module could be clearly discerned, consisting of a four-alpha helix bundle involving the N-termini of the corresponding subunits. The subsequent integrative structural model of the yeast exocyst, generated solely from low-resolution *in vitro* structural data [31], agreed well with the high-resolution cryo-EM structure.

Interactions with the yeast exocyst have been widely studied *in vitro*. Sec3 and Exo70 were found to have lipid binding domains, both binding phosphatidylinositol 4,5-bisphosphate (PI(4,5)P_2_) and anchoring the exocyst in the plasma membrane [32,33]. Exo70 was found to interact with small Rab guanosine triphosphatases (GTPases) Rho3 [34] and Cdc42 [35], and Sec3 with Rho1 [36] and Cdc42 [33,37], suggesting that these subunits are zones of regulation. Additionally, Sec3 binds the plasma membrane target-SNARE Sso1 and its paralog Sso2 [38], thereby promoting SNARE complex formation. Sec15 binds to the Myo2 motor protein associated with actin transport cables [39], as well as to the Rab GTPase Sec4 [40] and its GTP exchange factor Sec2 [41]. These three factors associate with the vesicle and regulate its tethering. Exo84 interacts with Sro7/77 [42], a beta-propeller tether that could be linking the exocyst's canonical tethering function with SNARE regulation. Sec6 interacts with fusion-related proteins, namely the vesicle-SNARE Snc1 and its paralog Snc2 [43], the target-SNARE Sec9 [44] and Sec1 from the Sec1/Munc-18 (SM) protein family [45].

The composition of the exocyst, its finer structure, and its interactions within itself and with the wider exocytosis protein community has been well defined in the field. For more detail on the isolated exocyst's low and high-resolution structure, and interactions, see the comprehensive review by Lepore et al. [46].

Open questions that remain largely concern its molecular mechanism. To answer, several techniques have been harnessed to push the context in which we can study the exocyst, within reconstituted *in vitro* systems, *in situ* cell systems, and *in situ* of the wider tree of life.

## Reconstituted: the exocyst between membranes

*In vitro* reconstitution is the building of a representative biological process using isolated components of that said process within a cell-free environment. It allows for determination of how molecular features drive biological outcomes [47]. Rossi et al. included the whole yeast exocyst complex in a membrane-reconstituted environment in 2020. This study, which utilized an *in vitro* assay that addressed homotypic tethering of post-Golgi vesicles isolated from secretory mutant strains, showed for the first time vesicle-to-vesicle tethering by the exocyst, in the presence of Sro7 and Sec4 [30] (Figure 1). An Exo70 gain-of-function mutant, which bypassed Rho3 and Cdc42 necessity, increased tethering efficiency. Negative stain EM of this mutant showed increased conformational flexibility of Exo70 and Sec6 (Figure 1), whose association with Sec9 [44] might lead to the transition between tethering and SNARE-mediated fusion. Thus, not only was the tethering function of the exocyst demonstrated, but its conformational dynamism mimicked *in vitro* to show that Rho3/Cdc42 are likely to induce a conformational change of Exo70 and Sec6 which could lead to increased Snc1/2 binding. A second component of yeast exocyst activation was investigated in a similar fashion [48], specifically the Sro7-mediated activation of the exocyst. Using *in vitro* reconstitution, the authors showed that the known interaction between the N-terminal domain of Exo84 and Sro7 [42] led to increased Sec4 avidity and vesicle-to-vesicle tethering, likely due a yet to be resolved global exocyst conformational change. Sro7-dependant activation is postulated to be functionally separate from the Rho3/Cdc42 activation of Exo70. *In vitro* reconstitution using the mammalian exocyst has also been achieved, with an emphasis on exocyst-lipid interactions. While yeast Sec3 and Exo70 bind plasma membrane PI(4,5)P_2_, but no direct binding to SV lipids has been shown [32,33], the mammalian exocyst directly binds PI(4,5)P_2_ in *trans*, enabling homotypic vesicle-to-vesicle tethering *in vitro* without the presence of Sec4 or Sro7 [49]. Recapitulation of the heterotypic tethering present *in vivo* (SV-to-plasma membrane tethering) remains as the next step towards reconstitution of the exocyst's mechanism of function.

Recently, *in vitro* reconstitution has suggested an emerging role of the yeast exocyst in promoting fusion. Combining a reconstituted membrane fusion system with Förster resonance energy transfer, the authors could detect conformational changes of the SNARE proteins by monitoring the proximity-related signal between two fluorophores tagged to different protein domains [50]. One of such SNAREs in yeast is Sso1. Its auto-inhibitory closed conformation and active open conformation, free to form the tertiary SNARE complex, were resolved by Munson et al. [51]. Lee et al. [50] were able to demonstrate *in vitro* that the exocyst induces the opening of this auto-inhibitory domain. This conformational switch increases the rate of Sso1 binding to Sec9, thereafter enhancing the formation of the *trans*-SNARE complex with Snc2. Exocyst addition also increased the population of fused vesicles. Whether additional components could yet exacerbate exocyst contribution to vesicle fusion remains to be explored.

## Structural cell biology: toward the exocyst in cells

Although reconstitution is powerful in direct association of molecular features with *in vitro* functions, other approaches are needed to characterize protein structure in a time-resolved native context. Much headway has been made in this regard concerning regulated exocytosis [52], however, constitutive exocytosis remains largely unexplored. In contrast to the triggering of regulated exocytosis by elevated calcium levels, the exocyst's highly dynamic behavior and lack of a known cellular regulatory switch for synchronization challenge the detection and analysis of constitutive exocytosis within the cellular context.

Live-cell imaging measurements paved the way towards better understanding the native conformation of the exocyst complex. Picco et al. [53] combined an *in situ* anchoring platform with localization microscopy to measure distances between a fluorophore fused to an exocyst subunit and a fluorophore tagged to the anchor. Distance measurements were done in 2D projection images with up to 2 nm precision. They did so for all orientations in which the complex can be anchored. Using the anchoring platform as a spatial reference to integrate the measured distances with known protein properties/interactions, they modeled the 3D architecture of the exocyst complex bound to a SV in living cells (Figure 1). The arrangement of rod-shaped subunits within modules had conformational agreement with the *in vitro* cryo-EM-based model, published a year later [11]. However, comparative analysis between models showed some organizational differences: Sec10 adopted a more rod-shaped conformation in contrast to a U-shape *in vitro* [54], the relevance of which is yet to be determined. Furthermore, a rotation of about 69° between the two tetrameric modules accounts for the main structural differences between the isolated and the *in situ* reconstructions, which provides a more ‘open’ conformation for the SV-bound exocyst [54]. Integrating the position of the SV (via Sec2-GFP) was instrumental for gaining contextual insight into this *in situ* architecture. Sec15 and Sec10 could be positioned closest to the vesicle, with Exo70 and Sec3 furthest away where the plasma membrane would define a plane tangential to the SV surface. Accordingly, the exocyst would be situated aside of the SV-plasma membrane interface (Figure 1), with Sec6 C-terminus pointing towards the open space near the SV-plasma membrane interface, a prime spot for facilitation of SNARE interactions and assembly.

Combining the near-atomic *in vitro* information of the cryo-EM model with the physiologically-relevant information from the *in situ* model has allowed a more functional high-resolution picture of the exocyst to emerge. Neither are able, however, to determine the high-resolution structure participating at native exocytic sites. Currently, this is likely only resolvable by subtomogram averaging (STA) of cryo-electron tomography (cryo-ET) data. Cryo-ET captures the *in situ* cellular context using electron-based imaging and 3D reconstruction, where complementation with (i) correlative fluorescence microscopy can provide protein identity information, and (ii) STA can resolve native protein structures at low-nanometer resolution. These worthy milestones are, however, not guaranteed to be attainable: the intrinsic dynamism of exocytosis (preventing functional synchronization), its characteristic short-lived events (reducing chance of capture) and the relatively low exocyst stoichiometry (low signal-to-noise ratio), challenge the analysis of exocytosis by *in situ* cryo-ET and subsequent STA of exocyst structure. So far, cryo-ET imaging of the exocyst has only been performed on the *in vitro* reconstituted mammalian complex (Figure 1). Maib and Murray [49] observed protein densities in between tethered membranes that had been incubated with purified exocyst. Interestingly, those membrane regions were consistently tethered at a distance of 32 nm, roughly the length of the cryo-EM exocyst model [11]. Thus, two conformational possibilities have been revealed: the exocyst in between the SV and plasma membrane, yielding 32 nm membrane separation as seen *in vitro*, or the exocyst positioned aside to the SV-plasma membrane contact interface (Figure 1), yielding minimal membrane separation, as proposed by Picco et al. [53]. The development of new fluorescent labels, imaging techniques and computational analysis tools will be vital for enabling future *in situ* cryo-ET validation of the exocyst's tethering position, as well as native high-resolution structure.

To achieve full mechanistic understanding, it also will be necessary to time-resolve the interplay of exocyst and its partners at exocytic sites. Using live-cell imaging and single-particle tracking, yeast exocyst was seen to travel with Sec4-positive vesicles (Figure 1), where after they co-localize on the plasma membrane for 18 s during tethering [55]. Importantly, it was demonstrated that the exocyst both arrives [56] and departs [55] as a holocomplex. This falls in contrast to previous reports by Boyd et al. [57] that differential temporal recruitment of exocyst subunits to the plasma membrane indicates control of exocyst localization by two of its subunits (Sec3 and Exo70). Dynamics not only indicate assembly and disassembly mechanisms, but also have the power to reveal temporal relationships with other exocytic proteins along the discrete functional steps of exocytosis. This is essential for validating *in situ* the roles that the exocyst plays during tethering and beyond. Through low expression of an exogenously tagged exocyst [58], it was possible to observe that clusters of the mammalian exocyst were present during SV fusion, up until the moment of complete fusion pore opening (Figure 1). Endogenous tagging of the mammalian exocyst using CRISPR-Cas9 was thereafter used to probe exocyst dynamics [59]. The two modules of the exocyst associate with the SV independently, rather than on the plasma membrane, and are both present by the time the vesicle arrives at the plasma membrane. Although independent in SV binding, both are required to complete exocytosis. Again, the exocyst was seen to remain up until the completion of fusion (Figure 1), with one discrepancy: Sec3 appeared to depart ∼1.7 s before other subunits.

The fluorescence detected by live-cell imaging is evidence that multiple exocysts contribute to each exocytic event. Although the higher-order mechanism that regulates their action could not be studied *in vitro*, *in situ* approaches open up the possibility to investigate how multiple copies of the complex are coordinated in time and space. The architecture in living cells presented by Picco et al. [53] suggests that up to 20 copies of the exocyst complex could arrange in a ring conformation around the SV, which would establish direct contact with the plasma membrane through the central hole of the ring. However, discrepancy remains about the exact exocyst copy number within individual events (Figure 1): while Picco et al. [53] measured 13 ± 1 Sec5 molecules in yeast exocytic events on the plasma membrane, Gingras et al. [56] measured 33 ± 11 copies of Sec5. In mammalian cells, Ahmed et al. [59] determined that 9.8 ± 3.5 Sec5 molecules were seen to associate with SVs on average. Consolidation of the copy number as well as experimental determination of the spatial organization of these multiple exocysts has yet to be achieved. Super resolution imaging and cryo-ET are two techniques being uniquely harnessed for this purpose in other biological processes, and their future application might open new avenues towards *in situ* exocyst architecture visualization.

## Evolutionary biology: the exocyst across the kingdom of life

The exocyst is a protein complex present in almost all eukaryotic organisms in which it has been looked for [60]. Therefore, it must maintain as well as adapt itself in order to preserve its functionality in different environments. Studying the heterogeneity of exocyst within the spectrum of organisms and physical environments where it is found, is a powerful approach to understand its core functional mechanism.

The exocyst presence in the eukaryotic supergroups Amoebozoa, Opisthokonta, Excavata, Archaeplastida and SAR (Stramenopiles, Alveolata and Rhizaria), and conserved CorEx motif, shows it has ancient origins before the last eukaryotic ancestor [60,61]. Across eukaryotes, the exocyst tends to maintain all eight subunits, suggesting the octamer works as a unit [61] despite evolutionary dynamism of sequence composition. Sec15 and Sec6 display sequence divergence in vertebrates, suggesting a diversification of notable interaction sites. In a similar vein, Exo70's divergence could be linked to its role in exocyst interaction with the plasma membrane and therefore adaptation of initiation and localization of exocytosis. Exo70 has had extreme expansion in plants, with up to 23 gene copies in *Arabidopsis* spp. [62], compared to just one copy in yeast and mammals. Exo70 paralogues, present across and within species, might therefore be expressed at different times, in different cell types, or have differential functions [63]. Notably, the functional mechanism of the exocyst beyond Opisthokonta (animals and fungi) and Archaeplastida (plants) has been mostly neglected, leaving a massive space of mechanistic and functional biodiversity unexplored.

Building on comparative genomics, predictions of the exocyst structure is an inevitable future direction within the field, and could offer evolutionary perspectives on structure and function. With the advent of deep learning approaches (e.g. RoseTTAFold [64] and AlphaFold [65–67]) accurate prediction of protein 3D-structure and interactions based solely upon its 1D-amino acid sequence are possible. This presents a major breakthrough, albeit with some limitations on overall prediction confidence. In the yeast exocyst's case, its *in vitro* high resolution and *in situ* low resolution structures have been reconstructed. However, how mutations, protein paralogues and species differences change that structure, are open questions too vast to be tackled with current experimental approaches. So far, AlphaFold has been used to predict plant exocyst structures. In particular, the Exo70B1 allele in rice [27] and in *Arabidopsis* spp. [68], was modeled in order to analyze pathogen interaction site variability in Exo70 alleles.

**Figure 2.**
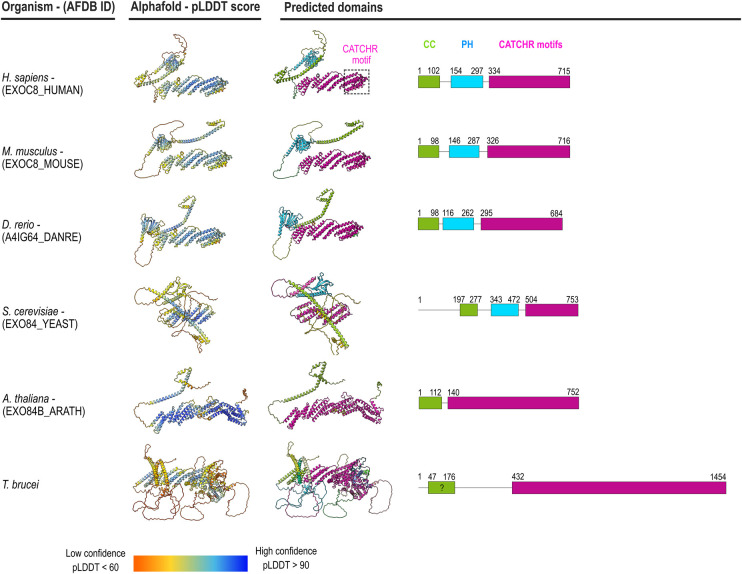
AlphaFold2 structure prediction for Exo84 subunit suggests structural similarities and differences. AlphaFold2 predicted structures along six Exo84 orthologs: *Homo sapiens*, *Mus musculus*, *Danio rerio*, *Saccharomyces cerevisiae*, *Arabidopsis thaliana* and *Trypanosoma brucei*. Predicted structures were downloaded from the AlphaFold Protein Structure Database [66,69] for *H. sapiens* (AFDB ID: EXOC8_HUMAN), *M. musculus* (AFDB ID: EXOC8_MOUSE), *D. rerio* (AFDB ID: A4IG64_DANRE), *S. cerevisiae* (AFDB ID: EXO84_YEAST) and *A. thaliana* (AFDB ID: EXO84B_ARATH). For *T. brucei*, Exo84 structure (not available in the AlphaFold Protein Structure Database) was modeled with the default AlphaFold Monomer v2.0 pipeline [66] using the sequence postulated in [70]. Structures on the left are color-coded according to the per-residue confidence score (pLDDT score) and those on the right according to the predicted domains (derived from the AlphaFold Predicted Aligned Error). The domain organization throughout the protein sequence is represented in the right panel. The high-confidence scored regions (pLDDT > 70, colored in blue) correspond to three structurally conserved domains: a C-terminal region containing CATCHR motifs (magenta), a Pleckstrin homology domain (PH, cyan), and a coiled-coil motif (CC, green), except *T. brucei* CC domain that has a pLDDT < 70. Structures were aligned to the CATCHR motifs using ChimeraX v.1.6 [83–85].

As an example of species-level application, we illustrate the utilization of AlphaFold2 [66,69] in predicting the structures of the Exo84 subunit across selected eukaryotic species from three supergroups: *Homo sapiens*, *Mus musculus*, *Danio rerio* and *Saccharomyces cerevisiae* from Opisthokonta, *Arabidopsis thaliana* from Archaeplastida and *Trypanosoma brucei* from Excavata (Figure 2). The structural organization of Exo84 is near identical for *H. sapiens*, *M. musculus* and *D. rerio* (species within the animal kingdom): a CC motif (98–102 residues) followed by a PH domain (141–146 residues) and a C-terminal region (381–390 residues) organized in antiparallel alpha helices grouped in three CATCHR motifs. For *S. cerevisiae*, the structural organization of Exo84 is slightly different: while the CC and the PH domains share similarity with the other Opisthokonta organisms, residues 1–196 are predicted to be unstructured or disordered (pLDDT score < 60) and the C-terminal region is reduced to two CATCHR motifs (249 residues). *A. thaliana* predicted Exo84 structure differentiates from the Opisthokonta organisms with the absence of the PH domain and an extended C-terminal region containing four CATCHR motifs (aa 140–752). However, a 112-residue CC motif at the N-terminus of the *A. thaliana* Exo84 resembles the previously mentioned organisms. Finally, for *T. brucei*, the protein sequence, almost double in length, seems to preserve the CATCHR motifs, which are intertwined by long disordered regions. Instead, the conservation of the N-terminal CC domain, characteristic of exocyst subunits as part of the CorEx motif, is controversial. Although the CC is not predicted by AlphaFold2 with confidence, a N-terminal long alpha-helix suggests that this feature might be present in *T. brucei,* a question that will need to be resolved experimentally. The PH domain was not predicted, diverging from the other supergroups. Thus, Exo84 predicted structures across species can show relative conservation, here characterized by CATCHR motif presence, but also differences, such as PH domain presence, number of CATCHR motifs and disordered regions. Comparative analysis has the potential to reveal core or heterogeneous structural features of the exocyst mechanism of function.

Structural predictions can expand upon what we know about the octameric exocyst complex, but fundamental changes are sometimes only detected by sampling life. Drastic examples of evolutionary change, such as total loss of the exocyst or gain of an extra subunit, although rare, tend to be associated with parasitic organisms. The gain of a subunit — Exo99 — to form a nonamer, occurred in kinetoplastids. This is a group of flagellated protists including many human parasites. Specifically, it was first detected and described as a component of the exocyst complex in *T. brucei* [70] and subsequently detected in *Leishmania* [71]. These species are responsible for African trypanosomiasis and leishmaniasis respectively, both deadly human diseases categorized as neglected tropical diseases [72]. The total loss of the exocyst complex has occurred in all Apicomplexa parasitic protists that have been sampled, including *Plasmodium falciparum* and *Toxoplasma gondii* [61]. It is thought that this could be due to the evolution of unique trafficking systems within this phylum, that could relieve the necessity of the exocyst [61]. It appears that significant evolutionary jumps involving the total loss of or add-ons to the exocyst are perhaps unique to parasites, suggesting an involvement in pathogenicity and a potential therapeutic target to manipulate.

Although not yet investigated in the pathogens themselves, their use of the host exocyst as a pathogen entry point is no new concept. This targeting of the host exocyst by pathogens is no more apparent as in plants. Plants require the exocyst for cellular defense against microorganism attack, such as for antimicrobial compound secretion and callose deposition in the cell wall [73]. This is illustrated by the accumulation of the exocyst at sites of invasion in live moss cells [74]. A plethora of viruses, bacteria and fungi have evolved mechanisms of host immune dampening by targeting the exocyst, and plants have responded by using the exocyst as a virulence detection molecule [75,76]. For example, *Magnaporthe oryzae* AVR-Pii effector binds to the rice exocyst subunit Exo70, presumably altering exocyst function by an unknown mechanism [77]. In response, the rice plant Pii gene is activated upon this binding event and triggers defense responses to halt pathogen invasion. The bacteria *Xanthomonas campestris* (causing black rot disease) XopP effector also binds Exo70 and disrupts its activity [78]. Thus, plant Exo70 is a meeting point of a kind of evolutionary ‘battleground’, which might be in part responsible for its particular paralogue expansion [76]. Beyond plants too: the potential co-opting of the mammalian exocyst for virus release was recently shown in the context of herpesvirus infection, where exocyst assembly is promoted to secrete virus-containing SVs [79]. With Exo70 silencing causing the largest reduction in viral release, this subunit's c-terminal PI(4,5)P2 binding residues were found to be necessary for the virus's secretion. On the opposite end of the infection timeline, *Salmonella* bacteria enter the mammalian cell using a mechanism involving the host exocyst [80,81] where it is hypothesized to be exploited in membrane pocket enlargement for bacteria engulfment. Inversely, bladder epithelium host cells can utilize their exocyst for expulsion of invading bacteria, acting as an immune response [82].

The exocyst's essentiality and multi-functionality makes it an evolutionary ‘hotspot’, highlighted by its role in host-pathogen interactions across species. Comparative genomics (and potentially structure prediction) has uncovered evolutionary patterns across the wider tree of life, and cell biology has been used to interrogate exocyst roles within individual species. The future might hold a space for an integrative approach, where cell biology can be used in a more systematic way to directly compare exocyst mechanisms across species in evolutionary time.

## Conclusion

A new front of cross-context research has begun, where well-established *in vitro* structural insights on the exocyst are being complemented. Aided by new technical developments, *in situ* structural information and evolutionary scenarios will begin to unearth the molecular mechanism governing exocyst multifunctionality. To fully achieve this goal, the future will likely have emphasis on application of quantitative imaging for *in situ* visualization of functional exocyst structure, predictive structural biology, and evolutionary cell biology approaches for interrogation of the exocyst core mechanism across the increasingly-sampled kingdom of life.

## Perspectives

- The exocyst is a hetero-octameric protein complex conserved in nearly all eukaryotes, whose role of linking vesicles with the plasma membrane and readying them for fusion, is constitutive, highly rapid, and involves structural dynamism.
- The exocyst structure and biochemical interactions have been solved *in vitro* across the past three decades with considerable detail, along with initial integration of *in situ* information.
- The molecular mechanism of exocyst function remains unsolved. *In situ* contexts to study the exocyst within will be needed to fully elucidate it. Particularly, *in situ* techniques will need to be applied to capture exocyst structure and biophysical behavior within the cellular-context. Expansion of model organisms and environments within which the exocyst is studied will be necessary for unbiased characterization of its core mechanism and adaptive potential.

## Abbreviations

CATCHR: complexes associated with tethering containing helical rods
CC: coiled coil
CorEx: Core of Exocyst
CRISPR: clustered regularly interspaced short palindromic repeats
cryo-EM: cryo-electron microscopy
cryo-ET: cryo-electron tomography
EM: electron microscopy
GFP: green fluorescent protein
GTPases: guanosine triphosphatases
GTP: guanosine triphosphate
PH: pleckstrin homology
PI(4,5)P_2_: phosphatidylinositol 4,5-bisphosphate
SNARE: Soluble N-ethylmaleimide-sensitive factor Attachment Protein Receptor
STA: subtomogram averaging
SV: secretory vesicle
